# Are required pre-treatment dental evaluations in the osteoporosis population a barrier to antiresorptive therapies?

**DOI:** 10.1016/j.bonr.2026.101926

**Published:** 2026-05-29

**Authors:** Angelina Villain, Marie-Charlotte Trojani, Christian Hubert Roux, Catherine Pesci-Bardon, Isabelle Prêcheur, Véronique Breuil

**Affiliations:** a Service de rhumatologie, Université Côte d'Azur, Centre Hospitalier Universitaire, Hôpital Pasteur 2, Nice, France; b Laboratoire PHEN-X, équipe CARE, UMR E4320 UniCa/CEA, France; c Laboratoire Micoralis EA 7354, Faculté de Chirurgie Dentaire, Université Nice Côte d'Azur, 06000, Nice, France; d Pôle Odontologie, Centre Hospitalier Universitaire, 06300, Nice, France

**Keywords:** Osteoporosis, Dental care, Bisphosphonates, Teleconsultation

## Abstract

**Purpose:**

Dental evaluation is recommended before bisphosphonates (BPs) initiation because of the rare but serious risk of medication-related osteonecrosis of the jaw. In this context, the objective of this study was to assess the prevalence of the indication for dental care (DC) prior to BPs initiation for osteoporosis (OP).

**Methods:**

Monocentric retrospective study between January 2019 and August 2022. Inclusion criteria: patients requiring OP treatment with dental evaluation (panoramic dental X-ray (PD) and dental teleconsultation). Exclusion criteria: PD without tele-dental evaluation, BPs for other indications than OP, OP not treated with BPs. Collected data: demographic characteristics, medical history, bone status, conclusions of tele-dental expertise.

**Results:**

130 patients were included, mean age of 74.3 years (±12.2), 102 were women. DC was required for 95/130 patients (DC group). In the DC group, 77/95 required surgical DC, 35/77 completed the surgical DC, and 30/35 initiated BPs. 18/95 patients required non-surgical DC, 6/18 completed the DC, and 5/6 initiated BPs. BPs were initiated in 35/95 (36,8%) patients in the DC group and 29/35 (82,9%) in the non-DC group.

**Conclusion:**

The prevalence of DC required before BPs initiation in OP patients is high and have a negative impact on the effective implementation of OP treatment. These results emphasize the need for healthcare policies aimed at improving access to DC and the necessity for clear guidelines regarding DC management in the context of BPs therapy.

## Highlights

• Dental teleconsultation showed that most osteoporotic patients needed care before bisphosphonates traitement.
• Although dental surgery was frequently required, completion rates remained low.
• Bisphosphonate initiation rates were significantly lowered by dental care requirements.
• One major obstacle to the delivery of osteoporosis treatment is unmet dental care.
• The findings emphasize the necessity of more precise procedures and standards for pre-therapeutic dental care.

## Graphical abstract

**Figure:**
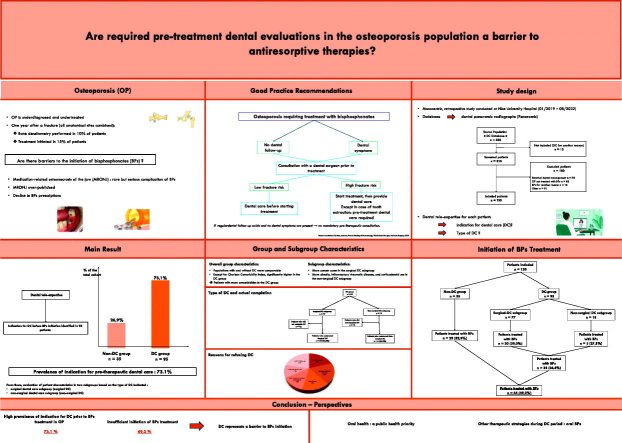

## 1 Introduction

Osteoporosis (OP) is a major public health issue due to its high prevalence and severe complications, such as fractures that increase morbidity and mortality. OP is responsible of a significant human and economic burden through loss of autonomy and high healthcare costs (Moayyeri et al., 2023; Sopina et al., 2025).

Bisphosphonates (BPs) are the most largely molecules prescribed for OP treatment and have revolutionized the management of the disease (Compston et al., 2019). Numerous randomized controlled trials have demonstrated their ability to increase bone mineral density (BMD) and reduce the risk of hip and vertebral fractures by 40% and 70%, respectively (Black et al., 1996; Eastell et al., 2019).

Despite this, OP remains largely underdiagnosed and undertreated (Zaheer and LeBoff, 2000; Miller, 2016; Roux and Briot, 2018; Cordova et al., 2024). Indeed, one year after an osteoporotic fracture, regardless of anatomical site, only 10% of patients have Dual-energy X-ray Absorptiometry, and only 15% received effective OP treatment in France (Briot et al., 2018a).

One of the main barriers to BPs treatment implementation is the risk of medication-related osteonecrosis of the jaw (MRONJ) (Amigues et al., 2023). MRONJ is a rare but serious complication of BPs, with an incidence of 2 to 5 per 100,000 patient-years for oral BPs and 9.6 per 100,000 patient-years for zoledronic acid (Ruggiero et al., 2022). The risk of BPs induced MRONJ received widespread media attention, responsible of fears in patients and in healthcare professionals (Cipriani et al., 2018). It has been reported that BPs prescription decreased following this media coverage (Amigues et al., 2023). Concerns about the long-term safety of OP treatments are among the main reasons why patients either do not initiate BPs therapy or discontinue it (Roux and Briot, 2018).

To date, the risk factors for MRONJ are well known (dental extractions, advanced age, female gender, diabetes, smoking, alcohol consumption, corticosteroid use, and chemotherapy) (Amigues et al., 2023; Ruggiero et al., 2022; Gunepin et al., 2013; Anastasilakis et al., 2022). In this context, French guidelines for good practice have been established years ago concerning oral health for patients with OP who are being treated or are to be treated with BPs (Société Française de Stomatologie, Chirurgie Maxillo-Faciale et Chirurgie Orale, 2013). It is recommended that patients with OP requiring BPs who lack rigorous dental follow-up undergo a pre-therapy dental evaluation, along with necessary treatment (Société Française de Stomatologie, Chirurgie Maxillo-Faciale et Chirurgie Orale, 2013; Laroche, 2024). Nevertheless, to the best of our knowledge, no study has assessed the prevalence of dental care (DC) indications prior to BPs in OP.

Thus, we undergone a retrospective study to evaluate the prevalence of DC required before BPs treatment in OP and the impact on BPs initiation. The primary objective was to assess the percentage of patients requiring DC before BPs initiation in osteoporotic patients. The secondary objectives were to evaluate (i) the characteristics of patients with an indication for DC compared to those for whom no DC was required, (ii) the percentage of patients who completed the recommended DC, and (iii) the percentage of patients who ultimately received BPs.

## 2 Methods

### 2.1 Study design

We conducted a monocentric retrospective study in the rheumatology department of the CHU of Nice between January 1, 2019, and August 1, 2022. Inclusion criteria: All adult patients who had a panoramic dental X-ray (PD) in the rheumatology department within the defined interval were included (PD database, CCAM code HBQK002). Non-inclusion criteria: Patients who underwent a PD for a reason other than a pre-therapeutic OP assessment (such as an assessment for endocarditis, septic arthritis, spondylodiscitis, or other causes) were not included. Exclusion criteria: (i) Patients who underwent a PD without teleconsultation because they were managed by their regular dentists; (ii) Patients with OP being treated with a therapy other than BPs; (iii) Patients who received BPs for a condition other than OP (Paget's disease, hypercalcemia, bone metastases).

### 2.2 Ethical considerations

The patients' consent for the use of their medical and personal data, including their imaging, was obtained upon their admission to the rheumatology department (either for conventional hospitalization, day hospitalization, or fracture care). This study was registered with ClinicalTrials.gov (registration number: NCT05719818).

### 2.3 Data collection

Data were collected retrospectively through medical record review (both paper and electronic records). If data were missing, patients were contacted by phone afterward. If missing data could not be retrieved, a “worst-case scenario” approach was applied. This approach was defined as follows: (1) when it was unknown whether dental care was indicated, it was assumed to be indicated; (2) when it was unknown whether the indicated dental care had been performed, it was assumed not to have been performed; (3) when the nature of the indicated dental care (surgical vs non-surgical) was unknown, it was assumed to be surgical; and (4) when it was unknown whether BPs therapy had been initiated, it was assumed not to have been initiated.

### 2.4 Clinical and biological data

For each patient, the following clinical data were collected: age, sex, weight, height, medical history including cardiovascular events (stroke and/or transient ischemic attack and/or myocardial infarction and/or peripheral arterial occlusive disease), respiratory, cancer, endocrine disorders, inflammatory rheumatism), substance use (alcohol, tobacco), corticosteroid therapy, age at menopause, DEXA results, history of low-energy fractures, and surgical or interventional management for fractures. Based on the collected clinical data, the Charlson Comorbidity Index (CCI) was calculated, with a significance threshold of a score strictly greater than 4, corresponding to an 85% risk of mortality within one year (Charlson et al., 1987). We also recorded the indication for starting OP treatment with BPs and the time between the indication and the actual initiation of BPs. After starting BPs, the data collected included medication adherence, the occurrence of new fractures, dental complications, and the need for DC during treatment.

For each patient, the following biological data were collected: corrected calcium levels, phosphorus levels, parathyroid hormone (PTH), 25(OH) vitamin D, and renal function (estimated by MDRD).

### 2.5 Data on dental teleconsultation

As part of the fracture pathway or during hospitalization in the rheumatology department of the CHU of Nice, patients included in the study underwent a dental teleconsultation. This teleconsultation included the performance of a PD and the transmission of clinical information to a dental surgeon specialist (IP), who then decided whether DC was indicated. The dental treatments were subsequently scheduled. We collected the following dental data: indications for DC (yes/no), types of DC indicated, and whether the DC was provided (yes/no).

### 2.6 Statistics

Descriptive analyses were performed using Medistica (pvalue.io, a Graphic User Interface for the R statistical analysis software for scientific medical publications. 2019–24. Available at: https://www.pvalue.io/fr). Continuous variables are expressed as means (± standard deviation), and categorical variables as absolute numbers and relative frequencies. Depending on the data distribution, groups were compared using the Chi-squared test, Fisher's exact test, or Welch's *t*-test. A *p*-value of less than 0.05 was considered statistically significant for all analyses.

## 3 Results

### 3.1 Patient characteristics (Fig. 1, Table 1)

**Fig. 1:**
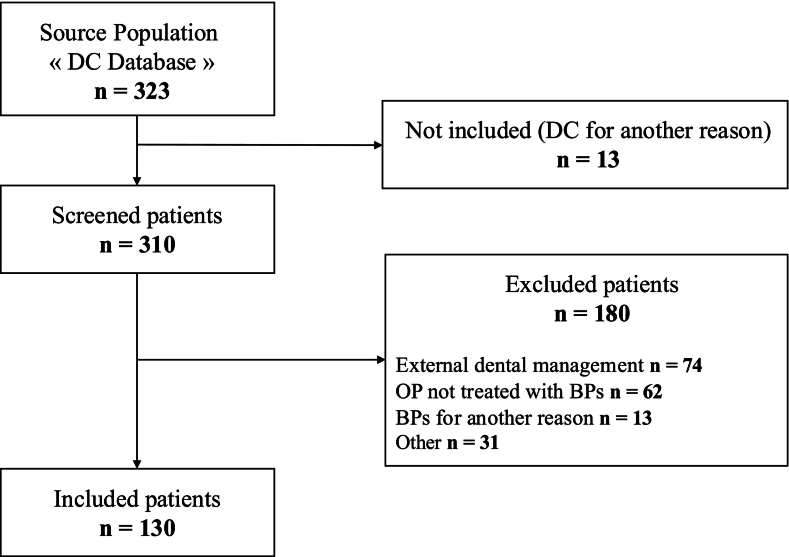
Study flowchart.

Among the 323 patients who underwent a PD in the rheumatology department of the CHU of Nice during the study period, 310 patients had the PD as part of a pre-therapeutic assessment for OP and were screened. One hundred eighty patients were excluded for the reasons summarized in the flow chart (Fig. 1). In total, 130 patients who underwent a PD as part of a pre-therapeutic OP assessment with an indication for BPs treatment were included. Each patient received a dental teleconsultation.

**Table 1:** Clinical and paraclinical characteristics of the total study population and by subgroups according to the indication for dental care (DC group) or not (non DC group).

Characteristics	Total Population (n = 130)	DC Group (n = 95)	Non-DC Group (n = 35)	p
Mean Age (± SD, [min - max])	74.3 (12.2, [41.0–97.0])	74.3 (11.6, [41–96])	74.1 (13.7, [45–97])	0,93
Sex Female, n (%) Male, n (%)	102 (78.5) 28 (21.5)	76 (80) 19 (20)	26 (74) 9 (26)	0,48 -
Mean Age at Menopause (± SD)	48.9 (5.6)	48.5 (5.93)	50.2 (4.40)	0,22
BMI < 19, n (%)	12 (9.2)	9 (9.5)	3 (8.6)	1
BMI > 30, n (%)	14 (10.8)	11 (11.6)	3 (8.6)	0,76
Cardiovascular History	66 (50.8)	49 (51.6)	17 (48.6)	0,76
Diabetes, n (%)	16 (12.3)	14 (14.7)	2 (5.7)	0,23
Solid Cancer, n (%)	27 (20.8)	22 (23.2)	5 (14.3)	0,27
Hemopathy, n (%)	10 (7.7)	4 (4.2)	6 (17.1)	0,23
MGUS, n (%)	6 (4.6)	5 (5.3)	1 (2.9)	1
Chronic Inflammatory Rheumatism, n (%)	26 (20)	19 (20)	7 (20)	1
Chronic Kidney Disease Stage 3, n (%)	15 (11.5)	13 (13.7)	2 (5.7)	0,35
Asthma and/or COPD and/or CRF, n (%)	24 (18.5)	16 (16.8)	8 (22.9)	0,43
Smoking (current or former), n (%)	42 (32.3)	33 (34.7)	9 (25.7)	0,33
Chronic Alcoholism, n (%)	13 (10)	10 (10.5)	3 (8.6)	1
Corticosteroid Therapy (> 7.5 mg/d for >3 months), n (%)	23 (17.7)	17 (17.9)	6 (17.1)	0,92
Charlson Comorbidity Index >4, n (%)	63 (48.5)	43 (45.3)	8 (22.9)	0,02
Vertebral Fracture, n (%)	66 (50.8)	47 (49.5)	19 (54.3)	0,63
Proximal Femur Fracture, n (%)	39 (30)	28 (29.5)	11 (31.4)	0,83
Pelvic Fracture, n (%)	6 (4.6)	2 (2.1)	4 (11.4)	0,045
Proximal Humerus Fracture, n (%)	8 (6.2)	7 (7.4)	1 (2.9)	0,68
Mean Lumbar Spine old (± SD)	0.819 (0.174)	0.819 (0.188)	0.820 (0.132)	0,98
Mean Femoral Neck BMD (± SD)	0.588 (0.106)	0.587 (0.109)	0.590 (0.098)	0,89
Mean Total Hip BMD (± SD)	0.733 (0.132)	0.733 (0.130)	0.731 (0.137)	0,94
Mean Lumbar Spine T-score (± SD)	−2.0 (1.6)	−2.01 (1.74)	−1.89 (1.16)	0,68
Mean Femoral Neck T-score (± SD)	−2.5 (1.6)	−2.38 (0.876)	−2.81 (2.66)	0,37
Mean Total Hip T-score (± SD)	−1.7 (1.0)	−1.68 (1.02)	−1.74 (0.959)	0,76
Mean Calcium Level, mmol/l (± SD)	2.32 (0.149)	2.32 (0.149)	2.32 (0.150)	0,96
Mean Phosphorus Level, mmol/l (± SD)	1.10 (0.207)	1.10 (0.189)	1.09 (0.253)	0,79
Mean PTH Level, ng/l (± SD)	57.0 (29.8)	59.3 (30.3)	50.1 (27.5)	0,21
Mean 25-OH Vitamin D, ng/ml (± SD)	32.3 (19.5)	32.2 (20.9)	32.6 (15.1)	0,89

BMD: Bone Mineral Density; BMI: Body Mass Index; COPD: Chronic Obstructive Pulmonary Disease; CRF: Chronic Respiratory Failure; MGUS: Monoclonal Gammopathy of Undetermined Significance; PTH: Parathyroid Hormone. [1]: Stroke and/or transient ischemic attack and/or myocardial infarction and/or peripheral arterial occlusive disease. p = comparison between DC and non-DC groups.

The clinical characteristics of the patients included are summarized in Table 1. The population was predominantly female (78.5%) with a mean age of 74.3 years (±12.2).

### 3.2 Evaluation of bone status (Table 1)

The data assessing the bone status of the population are also summarized in Table 1. One hundred eight patients (83.1%) had experienced at least one fracture, of which 75 patients (57.7%) had experienced at least one severe fracture. Bone densitometry data were comparable between the groups, as were as the biological data.

### 3.3 Indication for dental care (Fig. 2, Table 1)

**Fig. 2:**
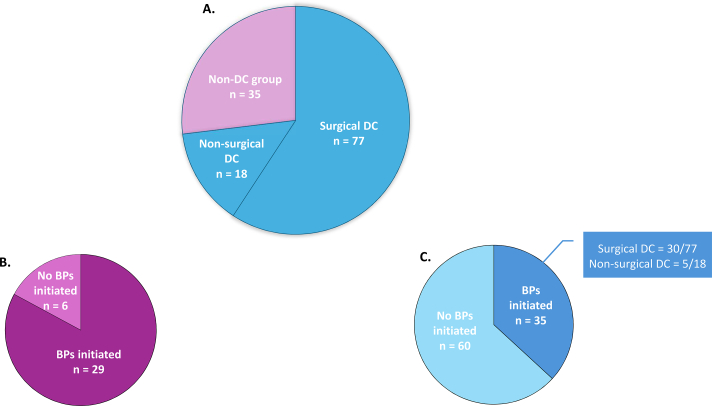
(in color): Distribution of dental care (DC) in the total population leading to subgroups definition. A. DC distribution in total population, *n* = 130; B. Number of BPs (bisphosphonates) initiation in Non-DC group, *n* = 35/130; C. Number of BPs initiation in DC group, *n* = 95/130.

Following the dental teleconsultation, the indication for DC of all types before initiating BPs was retained in 73.1% of cases (*n* = 95; DC group). Only 26.9% of patients required no DC (*n* = 35; non-DC group). Fig. 2 summarize the different subgroups.

The clinical and paraclinical characteristics of the total study population and of patients with and without an indication for DC are presented in Table 1.

### 3.4 Patient characteristics regarding the group

No statistically significant differences were found between the DC and non-DC groups for the various parameters collected, including the usual risk factors for MRONJ such as diabetes, smoking, corticosteroid exposure, and others. The only statistically significant differences were observed in the Charlson Comorbidity Index (CCI), which reflects patient comorbidities, and in the proportion of pelvic fractures. Indeed, the proportion of patients with a CCI strictly greater than 4 was significantly higher in the DC group compared to the non-DC group (*p* = 0.02). Additionally, the proportion of pelvic fractures was significantly higher in the non-DC group (*p* = 0.045).

### 3.5 Type of dental care (Table 2)

The different types of DC indicated included: dental extractions, dental caries treatments, abscess treatments, periodontal treatments, and endodontic treatments. These types of care can be divided into two categories: surgical DC, which includes dental extractions (at last one dental extraction), and non-surgical DC (treatments for dental caries, abscesses without extractions, periodontal treatments, and endodontic treatments).

**Table 2:** Clinical characteristics of patients in the DC group according to the type of DC indicated (surgical and non-surgical DC).

Characteristics	Surgical DC subgroup (*n* = 77)	Non-surgical DC subgroup (*n* = 18)	*p*
Mean Age (± SD, [min - max])	75.2 (11.2)	70.7 (13.1)	0,2
Sex Female, n (%) Male, n (%)	60 (78) 17 (22)	16 (89) 2 (11)	0,51 -
Mean Age at Menopause (± SD)	48.2 (6.52)	49.5 (3.61)	0.35
BMI < 19, n (%)	8 (10.4)	1 (5.6)	1
BMI > 30, n (%)	6 (7.8)	5 (27.8)	0,031
Cardiovascular History	39 (50.6)	10 (55.6)	0,71
Hypertension, n (%)	29 (37.7)	6 (33.3)	0,73
Cardiovascular Events [1], n (%)	17 (22.1)	3 (16.7)	0,76
Thromboembolic Events, n (%)	3 (3.9)	0 (0)	1
Heart Failure, n (%)	1 (1.3)	1 (5.6)	0,34
Atrial Fibrillation, n (%)	10 (13.0)	3 (16.7)	0,71
Diabetes, n (%)	12 (15.6)	2 (11.1)	1
Solid Cancer, n (%)	22 (28.6)	0 (0)	0,01
Hemopathy, n (%)	3 (3.9)	1 (5.6)	0,57
MGUS, n (%)	4 (4.7)	1 (11.1)	0,4
Chronic Inflammatory Rheumatism, n (%)	12 (15.6)	7 (38.9)	0,045
Chronic Kidney Disease Stage 3, n (%)	10 (13.0)	3 (16.7)	0,71
Asthma and/or COPD and/or CRF, n (%)	11 (14.3)	5 (27.8)	0,18
Smoking (current or former), n (%)	26 (33.8)	7 (38.9)	0,68
Chronic Alcoholism, n (%)	9 (11.7)	1 (5.6)	0,68
Corticosteroid Therapy (> 7.5 mg/d for >3 months), n (%)	10 (13)	7 (38.9)	0,017
Charlson Comorbidity Index >4, n (%)	37 (48)	6 (33.3)	0,26

BMI: Body Mass Index; COPD: Chronic Obstructive Pulmonary Disease; CRF: Chronic Respiratory Failure; MGUS: Monoclonal Gammopathy of Undetermined Significance. [1]: Stroke and/or transient ischemic attack and/or myocardial infarction and/or peripheral arterial occlusive disease. p = comparison between DC and non-DC groups.

Among the 95 patients with an indication for DC, 81.1% required surgical DC. The number of dental extractions required are available for 33/77 patients: median 2 (1 – 9). 18.9% required non-surgical DC. In the non-surgical DC subgroup, 4.2% had at least one dental abscess, 7.4% had an indication for endodontic treatment, 7.4% had at least one dental caries, and 8.4% had periodontitis.

The characteristics of patients were assessed based on the type of DC (surgical or non-surgical) and are summarized in Table 2.

### 3.6 Patient characteristics regarding the subgroup

Patients in the surgical DC subgroup had a higher incidence of solid cancers compared to patients in the non-surgical DC subgroup (*p* = 0.01). In contrast, the non-surgical DC subgroup included more patients with a BMI greater than 30 kg/m^2^, more patients with chronic inflammatory rheumatism, and more patients who had received oral corticosteroid therapy exceeding 7.5 mg per day for more than 3 months (*p* = 0.031, *p* = 0.045, and *p* = 0.017, respectively). No statistically significant differences were found between the two subgroups for the other parameters collected. However, the non-surgical DC subgroup was limited in size (*n* = 18), which reduces the statistical power of these subgroup comparisons and the differences between surgical and non-surgical DC subgroups should be interpreted with caution.

### 3.7 Provision of dental care

Among the 95 patients with an indication for DC, 41 (43.2%) received the recommended care. In the surgical and non-surgical DC subgroups, 45.5% (35/77) and 33.3% (6/18) of patients, respectively, received the indicated DC. Thus, 56.8% of the patients in the overall DC group did not undergo the indicated DC. As for surgical DC, 54.5% of patients did not undergo them. The reasons for not undergoing the recommended care were as follows: 7 patients considered the procedures too invasive, 6 patients discontinued them voluntarily, 11 patients cited financial reasons, 10 patients did not prioritize the treatment and therefore did not undergo it, 8 patients reported a lack of time, 2 patients passed away, and 10 patients were lost to follow-up.

### 3.8 Initiation of bisphosphonates treatment

Among the 41 patients who received the recommended DC, 35 (36.8%) were subsequently treated with BPs following their dental procedures. In total, 49.2% of patients who had an indication for BPs treatment (without distinction between the DC and no DC groups) received the treatment. It is noteworthy that in the non DC group, 82.9% (29/35) of patients were prescribed BPs treatment.

### 3.9 Type of bisphosphonate administered (Fig. 2)

68.8% were injectable forms (zoledronic acid), while 31.2% were oral forms (alendronate or risedronate). Fig. 2 summarize the DC subgroups.

## 4 Discussion

In this study, 73.1% of patients required dental care before initiating bisphosphonates for osteoporosis. To our knowledge, this is the first study to specifically address this issue.

Each year, approximately 40% of the general population undergoes DC, with between 5% and 7.9% requiring surgical DC according to various epidemiological studies (Ménard et al., 2016; Vincelet et al., 2008; Jabri and (DREES/SEEE/BACS), 2019). The prevalence of oral diseases in nursing home residents is 50%, with 26% requiring surgical DC, a significantly higher proportion than in other age groups (ORS Pays de la Loire, URPS chirurgiens-dentistes Pays de la Loire, 2019). The increasing prevalence of oral diseases in the elderly is partly explained by severe periodontitis, which can affect up to 66% of individuals aged 55 and older (Wiernik et al., 2024). Due to population growth and aging, the prevalence of these conditions is expected to continue rising in the coming decades, as projected through 2050 (Nascimento et al., 2024). According to the World Health Organization (WHO), poor oral health affects nearly half of the global population (45%, or 3.5 billion people), and it is estimated that 41% of French people do not receive regular dental follow-up (GBD 2017 Oral Disorders Collaborators et al., 2020; UFSBD / Webdentiste / Ipsos, 2013). In our study, the prevalence of patients requiring any type of DC appears to be significantly higher than in the general population. Several factors can explain this. First, the patients included were selected based on systematic dental evaluation, even among asymptomatic patients, which may have uncovered non symptomatic lesions. Additionally, the recruitment of patients from the rheumatology department at CHU de Nice, through fracture liaison service or conventional hospitalization, led to the selection of a cohort with severe OP, with 83.1% of patients having experienced a fracture, and 57.7% at least one severe fracture. As a result, the BPs prescribed were mainly injectable forms (68.8%). Lastly, 18% of patients were exposed to significant doses of corticosteroids. In this context, and due to the identified risk factors for MRONJ the indication for DC was broader (Amigues et al., 2023; Ruggiero et al., 2022; Gunepin et al., 2013; Anastasilakis et al., 2022). Therefore, our hospital-based cohort, characterized by severe OP and a substantial proportion of patients exposed to corticosteroids may not be representative of a typical outpatient being treated for OP. Consequently the 73.1% prevalence rate of required DC is probably higher than expected in a standard population. Lastly, the use of a worst-case scenario approach may have contributed to an overestimation of the prevalence of DC, particularly surgical DC.

Despite the significant annual need for DC in the general population in France, these procedures are still insufficiently performed. According to a 2018 study by the French Institute of Public Opinion (IFOP), 62% of French people reported having renounced DC at least once (Association Dentaire Française, 2018). The reported reasons for refusal were diverse: 45% due to the cost of treatment, 33% because of fear of pain, 31% due to difficulty obtaining an appointment within a reasonable timeframe, and 15% due to geographical distance (Association Dentaire Française, 2018). In our study, 56.8% of patients did not undergo the indicated SD for various reasons, including financial cost (20.4%), a sense of “non-priority” (18.5%), lack of time (14.8%), and fear of pain or the invasive nature of the SD (13.0%). These findings highlight the reasons for limited access to and the refusal of DC in France. The financial aspect, combined with the lack of reimbursement for some DC procedures by the French National Health Insurance (CPAM), complicates access for economically vulnerable populations, exacerbating social inequalities. According to IFOP, 63% of self-employed workers and 62% of executives and intellectual professions find it “easy” to access DC, compared to only 47% of manual laborers, with similar issues for middle-class workers (UFSBD / Webdentiste / Ipsos, 2013; Association Dentaire Française, 2018). Geographical accessibility to dental practitioners is also unequal across France, with rural areas being less well-served. This geographical barrier often compounds financial difficulties, as underserved regions tend to be economically disadvantaged (Bas, 2023). Furthermore, elderly populations face additional barriers to accessing DC, particularly due to mobility limitations and the lack of availability of dental professionals in nursing homes (UFSBD, 2019; Abbe Denizot et al., 2017).

This cohort, predominantly female (78.5%), with a mean age of 74.3 (±12.2) years, is consistent with the typical osteoporotic population (Briot et al., 2018b). Among the most common comorbidities, 34% of patients had high blood pressure, and 12% were diabetic, comparable to the prevalence of these conditions in the general population (Mills et al., 2020; Lovic et al., 2020). However, we observed that 18% of patients were exposed to oral corticosteroid therapy, a figure considerably higher than in the general population, where exposure ranges from 1% to 4.5% in menopausal women (Briot et al., 2014). This result can be explained by the high proportion of patients with chronic inflammatory rheumatism in this cohort (20% of patients). Regarding global comorbidities, analysis revealed that the Charlson Comorbidity Index was significantly higher in the DC group compared to the no DC group (*p* = 0.02), suggesting that patients requiring DC had more comorbidities. These comorbid patients are typically those for whom dental health becomes a secondary priority. Managing chronic conditions often requires ongoing attention with specialized consultations, which can push DC to the background (UFSBD, 2015). This helps explain the very high rate of non-performance of the recommended DC in this cohort (56.8%).

Despite the proven efficacy of BPs in reducing fracture incidence and achieving significant bone density gain, only 15% of patients receive anti-osteoporotic treatment one year after an osteoporotic fracture at any anatomical site (Briot et al., 2018b). This lack of treatment leads to increased healthcare costs associated with fractures, particularly due to the resulting loss of autonomy, institutionalization, and mortality rates, which can reach up to 24% within the year following a hip fracture (Briot et al., 2018b; HAS, 2017). Optimizing OP management is therefore a key public health issue, and it is essential to identify the limiting factors. Among patients in the surgical DC subgroup, only 39.0% initiated BPs, and the initiation rate was even lower in the non-surgical DC subgroup (27.8%). Overall, despite being treated in a specialized bone disease department, only 36.8% (35/95) of patients who needed DC received their BPs. In contrast, 82.9% of patients in the non-DC group initiated BPs. Thus, DC of any kind appears to be a barrier to the initiation of BPs. A total of 49.2% of the entire cohort (regardless of DC) started BPs. Although far from a satisfying score, it is higher than in the general OP population, further supporting the importance of structured expert pathways, where the initiation of anti-OP treatment is significantly higher compared to patients who do not receive specialized care (38.0% vs 17.2%) (Huang et al., 2023).

Taken together, these findings suggest that all types of DC, regardless of their nature, act as barriers to the initiation of BPs in OP indication. These observations should be interpreted in the context of evolving international guidelines. In our study, patients were recruited between 2019 and 2022, and indications for dental management prior to BPs initiation were based on the French national guidelines in force at that time (Société Française de Stomatologie, Chirurgie Maxillo-Faciale et Chirurgie Orale, 2013). Since the description of MRONJ in 2003 (Marx, 2003), successive international guidelines have progressively evolved toward a more nuanced, risk-based approach, moving away from systematically requiring dental treatment before therapy initiation. In particular, the 2022 update of the American Association of Oral and Maxillofacial Surgeons (AAOMS) recommendations by Ruggiero et al. emphasizes oral health optimization and prevention, while distinguishing oncology patients —with a high risk of MRONJ that requires stringent measures— from osteoporotic patients in whom the absolute risk is low and treatment initiation should not be unduly delayed (Ruggiero et al., 2022). Our data collection ended in 2022 and thus reflects clinical practice at the transition between these evolving recommendations, which may partly explain a relatively conservative approach to dental management in our cohort. This recent update opens the door to new strategies regarding the management of DC prior to the initiation of BPs therapy, with a greater emphasis on individualized, risk-adapted approaches.

In our study, we specifically assessed fear related to DC, but did not evaluate anxiety specifically linked to MRONJ itself. Concerns regarding MRONJ, often disproportionate to its very low incidence in patients treated for OP, may influence both patients' acceptance of treatment and physician's prescribing practices, contributing to delays in initiating antiresorptive therapy as well as reduced adherence. This aspect was not fully captured in our study, although it likely plays an important role in clinical decision-making. This highlights the need for improved education and clearer communication regarding the actual risk of MRONJ in OP.

Our study has several limitations. First, it is a retrospective study, which introduces a recall bias. Another inherent limitation of retrospective studies is the missing clinical and paraclinical data, along with a significant loss to follow-up. To address this, we applied the “worst-case scenario” method. Additionally, a lack of statistical power may have limited the ability to obtain statistically significant results in subgroup analyses. There is a selection bias, as patients from the source population with severe OP and risk factors MRONJ, such as corticosteroid exposure, were overrepresented. Furthermore, our study did not allow the assessment of patient-related factors that may influence treatment initiation, particularly the perception of MRONJ risk. Concerns about this complication, which may be overestimated in the context of OP, could represent an important barrier to BPs initiation independent of dental status itself. Due to the retrospective nature of our data, we were unable to evaluate the information delivered to patients, their understanding of the benefit–risk balance, or the role of shared decision-making in this context. This unmeasured, patient-driven component may therefore contribute to the delays observed. A further limitation to consider is that this study relied on a dental teleconsultation model based on panoramic dental X-ray combined with the transmission of clinical information to a dental surgeon specialist. In this context, no physical intraoral clinical examination or bitewing radiographs performed, which is associated with a known risk of missing some early pathologies (early caries, exact periodontal pocket depths, and early periapical radiolucencies). These diagnostic limitations are inherent to this tele-expertise model relative to a gold-standard clinical examination.

Overall, these limitations highlight the multifactorial nature of delays in BPs initiation, in which patient and clinician perception of MRONJ risk may also play an important role. Therefore, the challenge is to change the perception held by patients—and in some cases by physicians and dentists—regarding the very low risk of MRONJ compared with the benefits of OP treatment, a condition associated with increased morbidity and mortality.

To conclude, this study reports for the first time a high prevalence of pre-therapeutic dental care indications in a predominantly female, elderly osteoporotic population with a need for bisphosphonates. We observed a limited proportion of patients receiving the indicated dental care and initiating bisphosphonates in the dental care group compared to the non dental care group, suggesting that all types of dental care act as a barrier to the initiation of bisphosphonates. These findings emphasize the need for health policies aimed at improving access to dental care for patients. Indeed, oral health is intrinsically linked to overall health (UFSBD, 2019). Therefore, promoting oral health through prevention and improved access to care should be a public health priority, contributing to the overall well-being of the French population and to the management of osteoporosis. Moreover, this study underlines the necessity of clear and updated recommendations for dental care in the context of bisphosphonates treatment.

## CRediT authorship contribution statement

**Angelina Villain:** Writing – original draft, Methodology, Investigation, Formal analysis, Data curation, Conceptualization. **Marie-Charlotte Trojani:** Writing – original draft, Visualization, Supervision, Methodology, Investigation, Formal analysis, Data curation, Conceptualization. **Christian Hubert Roux:** Writing – review & editing, Validation. **Catherine Pesci-Bardon:** Writing – review & editing. **Isabelle Prêcheur:** Writing – review & editing, Methodology. **Véronique Breuil:** Writing – review & editing, Visualization, Validation, Supervision, Methodology, Conceptualization.

## Funding

This research did not receive any specific grant from funding agencies in the public, commercial, or not-for-profit sectors.

## Declaration of competing interest

The authors declare that they have no competing interests.

## Data Availability

Data will be made available on request.
